# Using LASSO regression to detect predictive aggregate effects in genetic studies

**DOI:** 10.1186/1753-6561-5-S9-S69

**Published:** 2011-11-29

**Authors:** Joel B Fontanarosa, Yang Dai

**Affiliations:** 1 Bioinformatics Program, Department of Bioengineering (MC 063), University of Illinois at Chicago, 851 S. Morgan Street, 218 SEO, Chicago, IL 60607-7052, USA

## Abstract

We use least absolute shrinkage and selection operator (LASSO) regression to select genetic markers and phenotypic features that are most informative with respect to a trait of interest. We compare several strategies for applying LASSO methods in risk prediction models, using the Genetic Analysis Workshop 17 exome simulation data consisting of 697 individuals with information on genotypic and phenotypic features (smoking, age, sex) in 5-fold cross-validated fashion. The cross-validated averages of the area under the receiver operating curve range from 0.45 to 0.63 for different strategies using only genotypic markers. The same values are improved to 0.69–0.87 when both genotypic and phenotypic information are used. The ability of the LASSO method to find true causal markers is limited, but the method was able to discover several common variants (e.g., *FLT1*) under certain conditions.

## Background

Recent advances have enabled researchers to study genetic associations with familial diseases in remarkable detail. Genome-wide association studies (GWAS) of common variants have revealed numerous genetic loci that significantly modulate phenotypes for a wide assortment of important clinical phenotypes, ranging from the expected risk of certain malignancies [1,2] to commonly measured clinical traits, such as lipid levels [3]. Nevertheless, it is increasingly evident that the common variants found in GWAS provide an incomplete picture of the underlying genetic risk for many of the familial diseases that have been studied [4-6]. Thanks to the increased availability of sequencing technologies and to large-scale efforts such as the 1000 Genomes Project, exome scans are becoming increasingly popular in complex disease genetics. These studies represent several new challenges in genetic analysis.

Although a variety of machine learning methods have been used in GWAS [7], penalized regression methods are among the most flexible and are thus well suited for analysis of data sets such as exome scans, which may contain both common and rare effects. Numerous penalized regression methods have been shown to be effective for both common and rare variants [4,8-10]. Zhou et al. [4] proposed a combination of group and least absolute shrinkage and selection operator (LASSO) penalties to find both rare and common variants using sets of markers grouped by pathway and gene. However, their method was evaluated using family breast cancer registry data, and its performance is unclear for larger scale data from GWAS.

To improve accuracy, some studies have imposed an arbitrary *p*-value cutoff to limit the number of genetic variants in the LASSO model [9], whereas others have applied the model across all variants using the LASSO penalty and a group penalty for the gene or pathway [4]. In this study, we propose an approach using a LASSO model that first selects sets of genetic variants for each pathway and gene and then generates an optimized LASSO model based on the selected marker sets. Taking advantage of information provided in the Genetic Analysis Workshop 17 (GAW17) exome data set, we can build two LASSO models for each pathway or gene based on regression on either disease status or a quantitative trait. This approach is more time-consuming than optimization of a LASSO model for the full set of variants. However, our strategy permits us to build individual optimal models on each variant set related to the pathway and gene, allowing a more flexible and accurate model determination. In the remainder of this paper, we examine the performance of this new approach using the GAW17 exome data set.

## Methods

### LASSO regression

We compare several LASSO models that incorporate gene, pathway, and phenotypic information in this study. For a response vector *Y* = (*y*_1_,*…*,*y_n_*) containing case-control labels coded as 0 or 1 for a set of *n* subjects, a genotype matrix *G* = (*X*_1_,…,*X_n_*), with each vector *X*_i_ consisting of *m* single-nucleotide polymorphisms (SNPs) coded as 0, 1, or 2, and a coefficient vector *β*, the standard logistic regression model:(1)

can be fitted using *Y* and *G*. However, this model is not well suited for large genetic studies with far more variables than samples, and it often results in inaccuracies as a result of model instabilities, colinearities, and overfitting. Several penalized regression methods have become popular in the analysis of large-scale genetic data sets [7,9] for their improved variable selection. In this study, we use the L_1_ LASSO penalty method, which selects *β* based on the maximization of:(2)

where *l*(*β* | *Y*, *X*) is the logistic log-likelihood and *λ* is the shrinkage parameter. The LASSO-penalized regression model can also be defined for a linear regression for a continuous response vector [11]. In this study, we evaluate several different strategies for applying a LASSO regression that incorporates gene, pathway, and phenotypic information into the model.

### Data description

The GAW17 data set contains 697 unrelated individuals from the 1000 Genomes Project genotyped at 24,487 autosomal SNPs from 3,205 genes [12]. Two hundred six pathways from the Kyoto Encyclopedia of Genes and Genomes (KEGG) [13] are represented, spanning 7,929 different SNPs and 1,100 different genes. We restrict our analysis to the 13,572 nonsynonymous variants in the study. Each of the 200 simulated data sets includes the following information for each individual: case-control status, three continuous quantitative traits (Q1, Q2, Q4), and three phenotypic features (Age, Smoking status, and Sex). We use a multidimensional scaling analysis based on genome-wide pairwise identity-by-state distances computed in PLINK [14] to determine three main continental population strata: African (Luhya, Luhya-additional, Yoruba-1, Yoruba-2, Yoruba-additional), Asian (Denver Chinese, Denver Chinese-additional, Han Chinese-1, Han Chinese-2, Han Chinese-additional, Japanese-1, Japanese-2, Japanese-additional), and European (CEPH-1, CEPH-2, Tuscan, and Tuscan-additional) [15,16]. We then generate three binary features to include in our model, assigning patients to their corresponding Asian, European, and African populations. Two main population outliers were removed from our analysis.

### Analysis

We use the R software package glmnet in our analysis for LASSO regression [11] and evaluate our models using a 5-fold cross-validation procedure for each simulation data set. More specifically, we split the data sets into five independent folds of approximately equal size such that the case-control ratios in each population are maintained in each fold. Models are trained using four folds of the data and then tested using the remaining fold. This procedure is repeated for each of the five training and testing fold combinations. To determine an optimal value λ* for each training set, we apply an inner loop of 10-fold cross-validation. Then λ* is used on the entire training set to build the final model for the evaluation of the testing fold. Finally, the averaged evaluation measures over the five testing folds are reported as the testing accuracy. In our analysis the evaluation measures are the area under the receiver operating curve (*A*_ROC_) for logistic models and the mean-square error for continuous linear regression models.

We consider three basic models: (1) LASSO logistic regression with all genetic variants included; (2) LASSO logistic regression for each of the (a) 3,205 genes or (b) 206 pathways, followed by a LASSO regression using the combined set of selected variants from all genes or pathways; and (3) three separate LASSO linear regression models for each of the continuous quantitative traits Q1, Q2, and Q4 for each pathway, followed by a LASSO logistic regression over the entire set of selected variants across all pathways.

For each of these strategies, we consider a genotypes-only model, a combined model that includes phenotype information (Age, Smoking, and Sex), and a restricted model that is limited to a fixed number of variables. In this study, the restricted models are limited to have a maximum of 50 variables.

Model 1 is similar to most other applications of the LASSO regression model, in which a single regularization parameter is used. This model is convenient and computationally efficient, but its ability to detect local effects within biologically meaningful subsets of genes that are of interest in an exome study may be limited. Models 2 and 3 first determine optimized models for each gene or pathway and then run a LASSO regression over the combined set of variants selected for each gene or pathway.

## Results

### Performance of the models

Results for all the models are shown in Table 1. Each of the 200 simulated data sets was analyzed separately. Because model 2 had a substantially longer running time, it was evaluated for only 50 (model 2a) and 150 (model 2b) randomly selected data sets. To determine the baseline performance for our models, we sampled several simulation data sets using 180 random variants (corresponding to the average size of the basic genotypes-only model 1 result). The expected average *A*_ROC_ for a randomly selected set of variants was 0.49. Similarly, we used glmnet to compute optimal models from the set of 160 causal simulation markers and determined that the average *A*_ROC_ of this optimal set of genotypes was 0.59. This value represents the average predictive accuracy of an optimized subset of the genetic variants responsible for assigning disease status in the simulation and is considered the target value of our models that use only genotype data. As observed in Table 1, the purely genetic models have *A*_ROC_ values closer to 0.55 for all models considered. The combined models with phenotypic features had an *A*_ROC_ of 0.82, a universally higher average testing *A*_ROC_ value independent of any genotypic combination. Because of the high marginal effect sizes of the phenotypic variables (Age, Sex, and Smoking status), these effects frequently overpowered the effect sizes of genetic markers included in the LASSO models. The unrestricted LASSO models often resulted in solutions with a large number of variables, limiting the practical utility of these models. The testing *A*_ROC_ values of the restricted models were often the same as or better than those of the unrestricted models, indicating better generalization ability for the restricted models. However, the predictive performance of the genetic component did not reach the best possible level, and the models included larger numbers of noncausal variants. The use of gene and pathway information did not result in meaningful improvements in the regression models with respect to predictive capability.

**Table 1:** Prediction results for various model types

Model	Model type	Training *A*_ROC_	Testing *A*_ROC_	Number of true^a^	Size^b^	*N*
1	Genotypes only	0.57	0.55	3.57	179.43	200
	Genotypes restricted	0.56	0.55	0.84	22.07	200
	Combined model	0.82	0.82	1.27	28.38	200
	Combined model restricted	0.82	0.82	1.06	18.70	200
2a	Genotypes only	0.61	0.54	9.98	545.33	50
	Genotypes restricted	0.56	0.55	0.86	21.66	50
	Combined model	0.83	0.81	2.78	94.32	50
	Combined model restricted	0.83	0.82	1.14	20.57	50
2b	Genotypes only	0.73	0.54	11.65	348.86	150
	Genotypes restricted	0.58	0.56	2.01	29.57	150
	Combined model	0.85	0.78	9.35	228.43	150
	Combined model restricted	0.83	0.82	2.48	29.26	150
3	Genotypes only	0.62	0.54	11.32	294.68	200
	Genotypes restricted	0.58	0.56	1.75	22.84	200
	Combined model	0.83	0.82	3.94	64.17	200
	Combined model restricted	0.83	0.82	2.04	20.40	200

^a^ Average number of causal simulation markers included.

^b^ Average number of variables in each model.

Averaged results from a 5-fold evaluation procedure on *N* simulation data sets. Training *A*_ROC_ values were obtained from the internal 10-fold cross-validation on the training sets, as implemented in the R package glmnet. Testing *A*_ROC_ values were determined by applying each of the trained models to the five independent testing sets.

### Variables selected by the models

Table 2 shows results from each experiment for the most frequent variables that were selected in at least four out of five trained models within a simulation data set for models 1 and 3. These results reveal that the true variants detected were predominantly common variants, but our model may also have some capacity to identify true rare variants. The gene- and pathway-based regression approaches did not seem to produce substantially different *A*_ROC_ values or find different casual variants than those found using the simpler LASSO approach. However, as shown in Table 2, the proportion of those casual variant occurring was higher in model 3, indicating a more robust model.

**Table 2:** Feature selection

Model type	Model 1						Model 3				
			
	Gene	SNP	Count^a^	MAF^b^	Causal^c^		Gene	SNP	Count^a^	MAF^b^	Causal^c^
Gene only	*FLT1*	C13S523	35	0.0667	Y		*FLT1*	C13S523	71	0.0667	Y
	*ADAMTS7*	C15S3360	22	0.0029	N		*SRPR*	C11S6885	63	0.0014	N
	*TG*	C8S4379	17	0.0050	N		*TG*	C8S4379	61	0.0050	N
	*MDN1*	C6S4146	15	0.0050	N		RPA3	C7S297	58	0.0007	N
	*GOLGA1*	C9S4013	13	0.0308	N		*LAMB3*	C1S10178	54	0.0007	N
	*FLT1*	C13S522	12	0.0280	Y		*RPL27*	C17S2981	52	0.0007	N
Gene restricted	*FLT1*	C13S523	19	0.0667	Y		*FLT1*	C13S523	44	0.0667	Y
	*TEX14*	C17S3819	9	0.0043	N		*FLT1*	C13S522	24	0.0280	Y
	*FLT1*	C13S522	8	0.0280	Y		*CYP3*A43	C7S2324	21	0.0976	N
	*UBA3*	C3S2197	7	0.0108	N		*TG*	C8S4379	18	0.0050	N
	*GOLGA1*	C9S4013	7	0.0308	N		*PRKCA*	C17S4578	16	0.1664	Y
	*CYP3A43*	C7S2324	7	0.0976	N		*PIK3C2B*	C1S9189	15	0.0065	Y
Combined	Age	Age	200	NA	Y		Age	Age	200	NA	Y
	Smoke	Smoke	163	NA	Y		Smoke	Smoke	185	NA	Y
	*FLT1*	C13S523	49	0.0667	Y		*FLT1*	C13S523	81	0.0667	Y
	*FLT1*	C13S522	16	0.0280	Y		*FLT1*	C13S522	34	0.0280	Y
	*PIK3C3*	C18S2492	7	0.0172	Y		*PIK3C3*	C18S2492	18	0.0172	Y
	*HFE*	C6S853	3	0.0036	N		*PRKCA*	C17S4578	8	0.1664	Y
	*ARNT*	C1S6533	3	0.0115	Y		*ARNT*	C1S6533	8	0.0115	Y
	*ACP1*	C2S1	2	0.0093	N		*UBA3*	C3S2197	7	0.0108	N
Combined restricted	Age	Age	200	NA	Y		Age	Age	200	NA	Y
	Smoke	Smoke	163	NA	Y		Smoke	Smoke	180	NA	Y
	*FLT1*	C13S523	49	0.0667	Y		*FLT1*	C13S523	75	0.0667	Y
	*FLT1*	C13S522	17	0.0280	Y		*FLT1*	C13S522	32	0.0280	Y
	*PIK3C3*	C18S2492	7	0.0172	Y		*PIK3C3*	C18S2492	17	0.0172	Y
	*ARNT*	C1S6533	3	0.0115	Y		*UBA3*	C3S2197	6	0.0108	N
	*LARGE*	C22S1540	3	0.0201	N		*ARNT*	C1S6533	6	0.0115	Y
	*MMS19*	C10S4869	3	0.0050	N		*KDR*	C4S1861	5	0.0022	Y

^a^ Number of times a given variable was observed in four out of five trained models.

^b^ Minor allele frequency.

^c^ Variables used to determine disease risk by the GAW17 simulators.

The top most frequent variables occurred in at least four out of five trained models for models 1 and 3. All models were run for the 200 simulation data sets.

## Discussion

In this paper, we assessed the utility of several different strategies for analyzing exome simulation data with a range of causal allele frequencies in the presence of quantitative and phenotypic information. A comparison of the three proposed approaches indicates that the simple LASSO regression model may be an efficient means to determine truly associated variants, but it must be modified to reduce the number of variables to avoid unreasonably large models and overfitting. As discussed in other studies of these data at GAW17, the primary genetic effects that were expected to be observed in this study were those from common variants, such as C13S523 and C13S522 in *FLT1*. As shown in Table 2, individual genetic variants were identified consistently in four out of five training models in only a minority of simulation analyses. For example, *FLT1* C13S523 occurred in at most 81 out of 200 simulations in the combined analysis for model 3. Some loss of power was expected in our analysis, because we developed our models using 80% of a simulation data set to obtain an independent evaluation of our methods’ predictive ability. However, if we consider the same model calculated on all 200 replicates using the entire set of patients (no training set), then *FLT1* C13S523 is included in 132 of 200 data sets. In larger studies or in studies that have a preexisting independent sample to validate the predictive model, this diminished power will not affect our method as strongly and our model may be better able to discern genetic predictors.

Some variants, for example, *PIK3C3*, appeared much more frequently in the models that combined genotypic and phenotypic effects than in models that considered only genotypes. To further investigate this finding, we built logistic regression models for *Y* and *PIK3C3*, adjusting for either only population variables or both population and phenotypic variables. *PIK3C3* was significant (*α* = 0.01) in 22 out of 200 data sets for the model adjusted for population only and in 105 out of 200 data sets for the model adjusted for both population and phenotypic variables, providing an explanation for this observation. Our analysis also indicates a significant relationship in the linear regression model for Q1 and *PIK3C3* adjusted for population only (184 out of 200 data sets) and adjusted for both population and phenotypic variables (197 out of 200 data sets) at *α* = 0.01. This may also explain the more frequent occurrence of *PIK3C3* in model 3 than in model 1 for the combined models.

Our method was able to reliably ascertain some true variants using subsets of the data for training. In addition, the signs of the regression coefficients for the frequently selected variants were highly consistent (about 99%) over different simulation data sets. However, the ability of our model to find true variants was also accompanied by a large number of noncausal variants. Because several long-range correlations exist within the GAW17 data set, a portion of the variants classified as noncausal in our study may actually be truly associated with the disease state or phenotypic traits. The predictive ability of the LASSO model using only genetic information is limited because none of the examined genomic subsets have a predictive ability that is comparable to that of the phenotypic variables. Nevertheless, incorporating these phenotypic variables into our model increases the proportion of causal genetic variants found using our method.

## Conclusion

Although our method is able to detect some causal rare variants, the results do not indicate that this is a promising approach for the general analysis of exome sequencing data that include causal rare variants. Identifying optimal sets of genetic variants for every gene and pathway in a data set may take considerably higher computation time than the standard LASSO model and is expected to generate robust predictive models only when there are several adequately powered common causal variants to distinguish case subjects from control subjects.

## Competing interests

The authors declare that there are no competing interests.

## Authors’ contributions

JF and YD collaborated on the design of the study. JF carried out the data analysis and drafted the manuscript. YD oversaw the project, participated in the data analysis, and helped to draft the manuscript. Both authors read and approved the final manuscript.
